# Smoking cessation reduces the lectin-like low-density lipoprotein receptor index, an independent cardiovascular risk marker of vascular inflammation

**DOI:** 10.1007/s00380-017-1026-z

**Published:** 2017-07-31

**Authors:** Maki Komiyama, Hiromichi Wada, Koh Ono, Hajime Yamakage, Noriko Satoh-Asahara, Sayaka Shimada, Masaharu Akao, Tatsuya Morimoto, Akira Shimatsu, Yuko Takahashi, Tatsuya Sawamura, Koji Hasegawa

**Affiliations:** 1 Division of Translational Research, Clinical Research Institute, National Hospital Organization Kyoto Medical Center, 1-1 Mukaihata-cho, Fukakusa, Fushimi-ku, Kyoto, 612-8555 Japan; 2 Division of Molecular Medicine, School of Pharmaceutical Sciences, University of Shizuoka, Shizuoka, Japan; 3 Department of Physiology, Shinshu University, Nagano, Japan

**Keywords:** Smoking, Smoking cessation, LOX index, LOX-1, Inflammation, Atherosclerosis

## Abstract

Vessel wall inflammation promotes the destabilization of atherosclerotic plaques. The lectin-like oxidized low-density lipoprotein (LDL) receptor-1 (LOX-1) expressed by vascular cells and monocytes. LOX index is calculated by multiplying LOX-1 ligand containing apolipoprotein B level with the soluble LOX-1. A high LOX index reflects an increased risk for stroke and myocardial infarction. However, the change in LOX index after smoking cessation and the relationship between smoking-related variables and LOX index are unknown. Relation of the clinical parameters to the LOX index was examined on 180 subjects (135 males and 45 females) at the first visit to our outpatient clinic for smoking cessation. The impact of smoking cessation on the LOX index was also determined in the 94 subjects (62 males and 32 females) who successfully stopped smoking. Sex-adjusted regression analysis and multivariate analysis identified three independent determinants of the LOX index, namely, low-density lipoprotein-cholesterol (LDL-C; *β* = 0.311, *p* < 0.001), high-sensitivity C-reactive protein (*β* = 0.358, *p* < 0.001), and expired carbon monoxide concentration reflecting smoking heaviness (*β* = 0.264, *p* = 0.003). Body mass index (BMI) significantly increased 3 months after the onset of smoking cessation (*p* < 0.001). However, the LOX index significantly decreased (*p* < 0.001), regardless of the rate of increase in BMI post-cessation. The LOX index is closely associated with smoking heaviness as well as dyslipidemia and an inflammation marker. Smoking cessation may induce a decrease in this cardiovascular risk marker, independently of weight gain.

## Introduction

The leptin-like oxidized low-density lipoprotein receptor-1 (LOX-1) is a cell surface receptor of atherogenic oxidized low-density lipoproteins (Ox-LDL) expressed by vascular endothelial cells and monocytes. LOX-1 was suggested to play a role in the destabilization of atherosclerotic plaques. Dysfunctional vascular endothelium cells overexpress LOX-1, and some of the receptors are released into the bloodstream in a soluble form (sLOX-1). A correlation was established between the serum levels of sLOX-1 and LOX-1 [1]. Circulating sLOX-1 is used as a biomarker of acute coronary syndromes [2]. On the other hand, LOX-1 ligand containing ApoB (LAB) is a modified LDL that binds to LOX-1. LAB is considered a better marker than the standard lipid parameters [3, 4]. Serum LAB levels predict the risk of cardiovascular events [5]. In addition, high LAB levels have been reported in smokers and patients diagnosed with the metabolic syndrome [6]. An overexpression of LAB stimulates the production of LOX-1. When LOX-1 binds to a modified LDL, chronic inflammation is induced in vascular endothelial cells, resulting in atherosclerosis.

The LOX index is calculated by multiplying the concentration of LAB and sLOX-1 [LOX index = LAB × sLOX-1]. The LOX index measures the risk level of vascular wall curing and disease progression during the initial stage of arteriosclerosis [7]. Low-density lipoprotein-cholesterol (LDL-C) is a predictor of atherosclerosis [8], but it is not a reliable marker of cardiovascular risk, because approximately 30% of the myocardial infarctions occur when the patient’s LDL-C level is within the normal range. Furthermore, there is no relationship between the incidence of stroke and LDL-C levels [9]. On the other hand, LOX index reflects the initial stage of arteriosclerosis when other markers are still in the normal range. As such, the LOX index was suggested to be a useful marker for the early diagnosis of stroke and myocardial infarction [7]. An 11-year prospective cohort study conducted on 2437 subjects revealed that a high LOX index reflects an increased risk of stroke (approximately three times) and myocardial infarction (approximately twice) [10]. Finally, the LOX index is used as a preventive tool to raise awareness by quantifying the future cardiovascular risk, and might, therefore, be useful for the development of preventive medicine.

Smoking is a significant risk factor of atherosclerosis and cardiovascular disease [11]. The prevalence of cardiovascular disease is three to four times higher among smokers than non-smokers [12]. The mortality rate from myocardial infarction and stroke increases proportionally with the number of cigarettes smoked per day [13, 14]. For Japanese men, the relative risk of death from heart diseases is 4.2-fold higher for smokers who consume less than 20 cigarettes, and 7.4-fold higher for smokers who consume more than 20 cigarettes, as compared with non-smokers [15]. Fortunately, the cardiovascular risk decreases within 2 years after smoking cessation [12, 13]. It has been reported that sLOX-1 levels are associated with an inflammatory marker, high-sensitivity C-reactive protein (hsCRP) in smokers [16]. However, no study has investigated the potential of the LOX index as a cardiovascular risk marker in smokers, and the relationship between the LOX index and smoking cessation. Therefore, the present study investigated the association between the LOX index and smoking-related factors, and the impact of smoking cessation on the LOX index.

The α1-antitrypsin-low-density lipoprotein complex (AT-LDL) is an oxidatively modified LDL that accelerates atherosclerosis. We previously reported that the decrease (improvement) in serum AT-LDL levels detected after smoking cessation is suppressed by weight gain after smoking cessation [17]. The AT-LDL levels decreased in the subjects with a low BMI increase, but not in those with a high BMI increase. However, the possible impact of weight gain after smoking cessation on the LOX index is unknown. Therefore, the present study addressed this question as well.

## Materials and methods

### Participants

A prospective study was conducted on Japanese smokers from April 2007 to March 2010. All subjects enrolled in this study consulted the Smoking Cessation Clinic located at the Health Evaluation Center National Hospital Organization of the Kyoto Medical Center. The exclusion criteria were as follows: an acute coronary syndrome; an infection or a pyrexial illness; a recent myocardial infection or stroke (<3 months); an extensive renal transplant or a serum creatinine level of ≥3 mg/dL; liver failure defined as a chronic hepatic disease (e.g., cirrhosis) or biochemical evidence of significant hepatic dysfunction (e.g., bilirubin level of >threefold higher than the upper limit of the normal range, in association with aspartate aminotransferase/alanine aminotransferase/alkaline phosphatase activity levels of >threefold higher than the upper limit of the normal range); and an active inflammatory diseases. A high TG level would decrease the reliability of the LDL-C level. Therefore, we excluded 27 individuals with a TG level of ≥400 mg/dL or for whom the TG level during their initial visit to the smoking cessation clinic remained unknown. An informed written consent was obtained from all participants. They were not coerced into taking part in this study. All study data were anonymized by the removal of personal identifiers. The Ethical Review Board, National Hospital Organization, Kyoto Medical Centre approved the study protocol.

### Smoking cessation clinic and data collection

All anti-smoking treatments were conducted according to the Standard Procedures for Anti-Smoking Treatment (originally issued in March 2006 by the Japanese Circulation Society, Japan Lung Cancer Society, and Japanese Cancer Association) [18]. The subjects were examined at their first visit, and then 2, 4, 8, and 12 weeks later. The treatment modalities were transdermal nicotine patches or the oral administration of varenicline. At each visit, the subjects were questioned to verify the maintenance of smoking cessation, and specific advice was given by a nurse and a doctor to facilitate smoking cessation. The smoking states of each subject were assessed at the end of the 12-week anti-smoking treatment and 1 year after the smoking cessation treatment. Abstinence was confirmed by the subject’s non-smoking statement and an expired carbon monoxide (CO) concentration of ≤7 parts per million (ppm). An attempt to quit smoking was deemed unsuccessful when the subject stopped attending the visits during the treatment period or attended the visits but failed to maintain smoking cessation.

Body mass index (BMI) was calculated as the weight expressed in kilograms divided by the height expressed in meters squared. Systolic blood pressure (SBP) and diastolic blood pressure (DBP) were measured in a sitting position, after a resting period of >5 min, using an automatic electronic sphygmomanometer (BP-103iII; Nippon Colin, Komaki, Japan) [19]. A regular sized cuff appropriate for Japanese individuals (arm length 17–32 cm) was used as recommended. At each visit, a nurse used an EC50 Micro Smokerlyzer^®^ (Bedfont Scientific, Ltd., Kent, UK) to measure electronically the end-tidal CO concentration with a reported precision of >98% [20]. During the first consultation, nicotine dependence was assessed using the Fagerström Test for Nicotine Dependence (FTND), a global standard test of the physical dependence on nicotine [21–23]. The scores range from 0 to 10, with the higher scores indicating more severe nicotine dependence. The number of cigarettes smoked per day was determined by asking the question: “on average, in the past month, how many cigarettes did you smoke per day?”

### Blood analysis

Blood analysis was conducted to monitor the biochemical and hematological profiles of the study participants. The blood samples were collected from the antecubital vein 2–3 h after lunch to measure the levels of hemoglobin A1c (HbA1c), high-density lipoprotein-cholesterol (HDL-C), LDL-C, and hsCRP. The blood samples were immediately centrifuged (3000 rpm; 10 min) at 4 °C. The plasma levels of HbA1c and the serum levels of HDL-C and LDL-C were measured using an automatic analyzer (LABOSPECT 008; Hitachi High-Technologies Co., Ltd., Tokyo, Japan) and enzyme-based reagents (Kyowa Medex Co., Ltd., Tokyo, Japan) [17]. The serum levels of LAB and sLOX-1 were measured using specific enzyme-linked immunosorbent assays (ELISAs) (Ikagaku Co., Ltd., Kyoto, Japan), as previously described [7]. Briefly, the LAB levels were measured with recombinant LOX-1, whereas the sLOX-1 levels were measured with a monoclonal anti-ApoB antibody (HUC20) and two monoclonal anti-human LOX-1 antibodies (TS92 and HCU5-40) [16].

### Statistical analysis

All statistical analyses were carried out using the Statistical Package for Social Sciences (SPSS) Statistics 17.0 (SPSS Inc., Chicago, IL, USA). Normality was assessed using the Shapiro–Wilk test. A logarithmic transformation of the CO and hsCRP levels was performed for the statistical analysis. The correlations between the LOX index and the smoking-related and atherosclerosis-related factors were examined by correlation analysis after adjustment for sex. The factors related to the LOX index were analyzed by multivariate analysis after adjustment for sex. Furthermore, the change in LOX index at 3 months after smoking cessation was examined using the paired *t* test for parametric data or the Wilcoxon signed-rank test with Bonferroni correction for non-parametric data. In addition, changes in data from before to after smoking cessation were compared by repeated-measures ANOVA between patients prescribed Nicotine patch and Varenicline. Statistical significance was set at *p* < 0.05.

## Results

### Clinical characteristics of the participants

Various parameters were evaluated in the 180 smokers (135 males and 45 females), aged 25–81 years (mean 60 ± 13 years), at our smoking cessation clinic. Table 1 shows the data collected during the first visit. The mean number of smoking years was 38 ± 12, median number of cigarettes smoked per day was 20 [20, 30], median CO concentration in exhaled breath was 16 [10, 24] ppm, and mean FTND score was 7.2 ± 1.9. In terms of medication, 73 subjects (40.6%) received anti-hypertensive agents, 36 (20.0%) subjects received statins, and 38 (21.1%) subjects received medications for diabetes mellitus.

**Table 1:** Clinical characteristics of smokers (*N* = 180)

Age (years)	60 ± 13
Male/female	135/45
BMI (kg/m^2^)	23 ± 4
SBP (mmHg)	128 ± 19
DBP (mmHg)	73 ± 12
HbA1c (NGSP) (%)	5.5 [5.2, 6.1]
HDL-C (mg/dL)	56 ± 17
LDL-C (mg/dL)	112 [89, 131]
hsCRP (mg/dL)	0.24 [0.14, 0.98]
Daily cigarette consumption (*n*)	20 [20, 30]
Smoking years	38 ± 12
CO (ppm)	16 [10, 25]
FTND score	7.2 ± 1.9

Data are presented as mean ± standard deviation or median [interquartile range]

### Correlations between the LOX index and the clinical parameters

Clinical correlation analysis revealed a positive relationship between the LOX index and LDL-C (*β* = 0.393, *p* < 0.001), log-transformed serum hsCRP (*β* = 0.257, *p* = 0.002), daily cigarette consumption (*β* = 0.175, *p* = 0.023), and the FTND score (*β* = 0.178, *p* = 0.018; Table 2). Multivariate regression analysis of these baseline data identified independent determinants of the LOX index, namely LDL-C (*β* = 0.311, *p* < 0.001), log-transformed serum hsCRP (*β* = 0.358, *p* < 0.001), and log-transformed expired CO concentration (*β* = 0.264, *p* = 0.003).

**Table 2:** Gender-adjusted analysis on correlation between LOX index and clinical parameters in smokers (*N* = 180)

	Univariate		Multivariate	
	*β* value	*p* value	*β* value	*p* value
Age (years)	−0.062	0.426	–	–
BMI (kg/m^2^)	0.137	0.068	–	–
SBP (mmHg)	0.005	0.948	–	–
DBP (mmHg)	0.088	0.250	–	–
HbA1c (%)	0.049	0.531	–	–
HDL-C (mg/dL)	−0.103	0.241	–	–
LDL-C (mg/dL)	0.393	<0.001	0.311	<0.001
Log_hsCRP	0.257	0.002	0.358	<0.001
Daily cigarette consumption (*n*)	0.175	0.023	–	–
Smoking years	0.015	0.853	–	–
Log_CO (ppm)	0.114	0.133	0.264	0.003
FTND score	0.178	0.018	–	–

*β* value: correlation coefficients, *R* ^2^ = 0.268

### The impact of successful smoking cessation on the clinical parameters

Table 3 compares the parameters collected at the first visit and 3 months after the onset of smoking cessation for the 94 participants (62 males and 32 females, mean 61 ± 12 years) who successfully quit smoking. At the first visit, the subjects reported a mean number of cigarettes smoked per day of 23 ± 11 and a mean number of smoking years of 39 ± 11, with an FTND score of 6.5 ± 2.3. The clinical examination revealed a significant increase in BMI (*p* < 0.001) and HDL-C (*p* < 0.001), and a significant decline in SBP (*p* = 0.010), CO concentration (*p* < 0.001), and LOX index (*p* < 0.001), from baseline to 3 months after the beginning smoking cessation therapy.

**Table 3:** Data on patients before and after 3 months of successful smoking cessation (*N* = 94)

	Baseline	3 months	*p* value
BMI (kg/m^2^)	23.6 ± 3.7	24.0 ± 3.7	<0.001a
SBP (mm Hg)	132 ± 16	127 ± 16	0.010a
DBP (mm Hg)	76 ± 11	76 ± 11	0.595a
HbA_1c_ (%)	5.4 [5.2, 5.8]	5.5 [5.2, 5.9]	0.163b
HDL-C (mg/dL)	55 ± 15	60 ± 17	<0.001a
LDL-C (mg/dL)	118 [87, 134]	118 [90, 140]	0.498b
hsCRP (mg/dL)	0.8 [0.3, 2.5]	0.7 [0.3, 2.4]	0.720b
CO (ppm)	13 [8, 19]	1 [1, 2]	<0.001b
LOX index	3239 [2216, 4865]	2480 [1568, 4065]	<0.001b

Data are presented as mean ± SD or median [interquartile range]

*p* value: a, paired *t* test; b, Wilcoxon signed-rank test

Of the 94 individuals who successfully quit smoking, one individual did not require a smoking cessation aid, whereas the others included 43 individuals who used a nicotine patch and 50 who used varenicline. Demographic characteristics prior to quitting smoking are shown in Table 4. Individuals who received varenicline consumed more cigarettes per day and had a higher FTND score (an indicator of nicotine dependence) than individuals who used a nicotine patch. In addition, changes in data from before to after smoking cessation were compared to determine differences related to the type of smoking cessation aid. The results of that comparison are shown in Table 5. Individuals who received varenicline experienced more increase in the LDL-C after smoking cessation than those who used a nicotine patch. However, changes in the LOX index from before to after smoking cessation did not significantly differ between the two groups.

**Table 4:** Clinical characteristics of smokers (*N* = 93)

	Nicotine replacement therapy (*n* = 43)	Varenicline (*n* = 50)	*p* value
Male/female	31/12	31/19	0.379
Age (years)	60 ± 14	61 ± 11	0.627
Daily cigarette consumption (*n*)	20 [15, 25]	20 [20, 25]	0.032
Smoking years	39 ± 12	40 ± 10	0.673
FTND score	5.9 ± 2.6	7.0 ± 2.1	0.035

Data are presented as mean ± standard deviation or median [interquartile range]

**Table 5:** Data on patients before and after 3 months of successful smoking cessation (*N* = 93)

	Nicotine replacement therapy (*n* = 43)			Varenicline (*n* = 50)			
	OM	3M	*p* value for group	OM	3M	*p* value for group	Time × group
BMI	23.8 ± 3.6	24.1 ± 3.6	0.038a	23.6 ± 3.7	24.0 ± 3.7	0.002a	0.505c
SBP	132 ± 14	128+16	0.043a	131 ± 18	127 ± 17.3	0.083a	0.941c
DBP	77 ± 12	76.1 ± 11	0.68a	76 ± 11	76 ± 10.8	0.688a	0.96c
HbAlc	5.4 [5.2, 5.7]	5.5 [5.2, 5.9]	0.387b	5.4 [5.1, 5.8]	5.4 [5.1, 6.0]	0.389b	0.284c
HDL-C	56 ± 16	57 ± 18	0.26a	55 ± 14	61 ± 17.4	<0.001a	0.027c
LDL-C	117 [87, 134]	118 [95, 143]	0.142b	119 [87, 135]	118 [88, 140]	0.808b	0.349c
hsCRP	0.6 [0.3, 2.4]	0.6 [0.3, 2.3]	0.646b	0.8 [0.4, 3.6]	0.9 [0.4, 3.0]	0.773b	0.582c
LOX index	2784 [1654, 5586]	2573 [1222, 4018]	0.003b	3440 [2 349, 4444]	2378 [1653, 4065]	0.018b	0.204c

Data are presented as mean ± standard deviation or median [interquartile range]

*p* value: a, paired *t* test; b, Wilcoxon signed-rank test; c, repeated measures ANOVA

Most individuals who visited the smoking cessation clinic but continued to smoke eventually stopped visiting the clinic. Accordingly, data from the initial smoking cessation clinic visit to 3 months after the initial visit (during the 5th visit) were compiled for only 18 individuals. Individuals who continued to smoke tended to exhibit a decrease in the LOX index from 11,595 (during the initial visit) to 6317 (after 3 months). However, these LOX index values were not significantly different (*p* = 0.145).

### The impact of weight gain after smoking cessation on the LOX index

Five participants were removed due to the lack of BMI data. For the remaining 89 subjects, the median rate of BMI increase was 1.297% from baseline to 12 weeks after the onset of smoking cessation therapy. This rate was used as the cut-off value to divide these 89 participants into two groups: those with a rate of BMI increase below the median (ΔBMI < median; 34 males and 10 females) and those with a rate of BMI increase at, or above, the median (ΔBMI ≥ median; 26 males and 19 females). Aside from the HbA1c level (*p* = 0.008), there was no significant difference in baseline data between the two groups.

Table 6 compares the data collected before and after smoking cessation for the ΔBMI < median group (upper part) and the ΔBMI ≥ median group (lower part). There was a significant decrease in LOX index 12 weeks after the onset of smoking cessation in both the ΔBMI < median group (28.1%; *p* = 0.001) and the ΔBMI ≥ median group (21.9%; *p* = 0.013). In addition, there was a significant increase in HDL-C (*p* = 0.002) and significant decrease in SBP (*p* = 0.029) in the ΔBMI < median group, but not in the ΔBMI ≥ median group.

**Table 6:** Patients’ data before and after 3 months of successful smoking cessation: comparison between patients with smaller versus larger BMI changes (*N* = 94)

	Baseline	3 months	*p* value
ΔBMI (%) < median^A^			
BMI (kg/m^2^)	23.8 ± 4.2	23.5 ± 4.1	**0.002**a
SBP (mmHg)	131.5 ± 17.6	126.6 ± 16.7	**0.029**a
DBP (mmHg)	75.0 ± 12.6	74.1 ± 11.5	0.483a
HbA1c (%)	5.6 [5.3, 6.4]	5.6 [5.2, 6.3]	0.782b
HDL-C (mg/dL)	55.7 ± 16.4	61.8 ± 17.1	**0.002**a
LDL-C (mg/dL)	112.6 [89.1, 133.0]	113.5 [88.1, 137.3]	0.703b
hsCRP (mg/dL)	0.8 [0.2, 3.6]	0.6 [0.3, 2.3]	0.156b
LOX index	3058 [1736, 5220]	2198 [1548, 3328]	**0.001**b
ΔBMI (%) ≥ median^A^			
BMI (kg/m^2^)	23.3 ± 3.2	24.5 ± 3.3	**<0.001**a
SBP (mmHg)	132.5 ± 15.1	128.5 ± 16.5	0.123a
DBP (mmHg)	78.4 ± 8.7	77.6 ± 9.7	0.525a
HbA1c (%)	5.3 [5.1, 5.6]	5.3 [5.1, 5.6]	0.075b
HDL-C (mg/dL)	54.3 ± 13.5	56.6 ± 18.0	0.072a
LDL-C (mg/dL)	122.1[87.0, 134.3]	118.5 [95.0, 142.0]	0.795b
hsCRP (mg/dL)	0.8 [0.4, 2.2]	1.0 [0.4, 3.9]	0.351b
LOX index	3517 [2269, 4940]	2748 [1650, 4477]	**0.016**b

Data are presented as mean ± SD or median [interquartile range]

Bold indicates statistical significance *p* values (*p* < 0.05)

*p* value: a, paired *t* test; b, Wilcoxon signed-rank test

^A^ Median ΔBMI = 1.297%

## Discussion

The LOX index is a biomarker used to predict the onset of myocardial infarction and stroke [7, 10]. In the present study, the multivariate regression analysis, conducted on the baseline data collected before the smoking cessation treatment, indicated that the LOX index was positively correlated with markers of dyslipidemia (LDL-C), inflammation (hsCRP), and smoking heaviness (exhaled CO concentration). The rate of mortality from myocardial infarction and stroke was reported to increase with the number of cigarettes smoked per day [13, 14]. Therefore, there may be a close relationship between the LOX index, smoking, and cardiovascular diseases.

The cardiovascular risk decreases within 2 years after smoking cessation [12, 13], but takes >10 years to reach the level of a non-smoker [24]. Studies showed that serum hsCRP levels return to the normal range within 5–10 years after smoking cessation. However, the present study revealed a significant decrease (improvement) in the LOX index from baseline to 3 months after the onset of smoking cessation therapy. Interestingly, LOX index has been shown to reflect the progression of arteriosclerosis in the early stage, when other markers are still in the normal range. Furthermore, the LOX index was suggested to be a useful marker for the early diagnosis of cardiovascular diseases [7]. In the present study, the LOX index decreased (improved) significantly within 3 months after smoking cessation. This finding suggests that the LOX index may contribute an early marker of the decrease in cardiovascular risk after smoking cessation. Individuals who visited the smoking cessation clinic but continued to smoke tended to have a lower LOX index. Among individuals who continued to smoke while visiting the smoking cessation clinic, the exhaled CO concentration decreased from 12 ppm during the initial visit to 5 ppm at 3 months after the initial visit. This reduction in smoking presumably accounted for tendency of the decrease in the LOX index.

Our study observed no difference in the extent of the decrease in the LOX index after smoking cessation related to the type of smoking cessation aid. However, the two groups differed with respect to the characteristics of individuals; for example, those who received varenicline consumed more cigarettes per day and had a higher nicotine dependence level. It is unclear why the extent of changes in HDL-C differs between varenicline and nicotine patch groups. A prospective, double-blinded randomized controlled trial must be conducted to rigorously compare the extent of changes in various parameters including the LOX index associated with the type of smoking cessation aid.

Although the cardiovascular risk decreases to a half at early stage after smoking cessation [12, 13], and thereafter, it decreases slowly [24]. The mechanisms regulating the rate of decline in the risk are unknown. Nonetheless, the inflammation caused by weight gain after smoking cessation might be one of the factors. We previously reported that the AT-LDL, which is known to accelerate atherosclerosis, decreased in the ΔBMI < median group but not in the ΔBMI ≥ median group after smoking cessation [17]. In contrast, the LOX index decreased in both groups at 3 months after smoking cessation. Regarding the extent, however, the LOX index decreased by 28% in the ΔBMI < median group and 22% in the ΔBMI ≥ median group after smoking cessation. Accordingly, the possibility that weight gain might attenuate this decrease (i.e., improvement) in the LOX index after smoking cessation cannot be ruled out. Moreover, the AT-LDL level has been associated with the number of smoking years [25] and, therefore, may be affected by smoking for a prolonged period. In contrast, the LOX index is related to exhale CO concentration, which reflects the smoking status in the past 24 h. In other words, the LOX index may be an acute indicator of inflammation that better reflects the recent smoking status than the duration of smoking (as reflected by the AT-LDL level). The LOX index is determined by multiplying the LAB level by the sLOX-1 level, which appears in the blood when LOX-1 is cleaved and released. Therefore, sLOX-1 levels in the blood reflect the expression of LOX-1, which increases 24 h after exposure to various types of stimuli. As a result, the LOX index may shift within a short period of time. In contrast, AT-LDL is an oxidized/modified LDL, and its level is affected by an increase in the LDL-C level as a result of weight gain. Therefore, AT-LDL is affected by slow changes in weight (e.g., 3 months) after smoking cessation, whereas the LOX index reflects acute inflammation due to smoking. The LOX index might thereby reflect the effectiveness of smoking cessation prior to weight gain.

A prospective study of individuals who underwent health checkups suggested that the LOX index is a future indicator of cerebral and myocardial infarction [10]. The current study observed a significant decline in the LOX index as a result of smoking cessation during a 3-month period. Nevertheless, whether a decline in the LOX index after smoking cessation leads to and correlates with a decrease in cardiovascular events must be studied further in the future.

There is a limitation to this study. The analysis only covered 3 months after smoking cessation. Therefore, other studies should be conducted to determine the long-term impact of smoking cessation and weight gain post-cessation on the LOX index. As the LOX index detected, with high sensitivity, the change in smoking states early after cessation, further studies should assess whether the LOX index could be used to grade the effects of short-term smoking reduction and side-stream smoke.

## Conclusions

The LOX index is closely associated with recent smoking states as well as dyslipidemia and an inflammation marker. Smoking cessation may induce a decrease in this cardiovascular risk marker, independently of weight gain.

## Abbreviations

LDL: Low-density lipoprotein
LOX-1: Lectin-like oxidized low-density lipoprotein receptor-1
sLOX-1: Soluble LOX-1
LAB: LOX-1 ligand containing ApoB
LDL-C: Low-density lipoprotein-cholesterol
CO: Carbon monoxide
ppm: Parts per million
BMI: Body mass index
SBP: Systolic blood pressures
DBP: Diastolic blood pressures
FTND: Fagerstrom test for nicotine dependence
HbA1c: Hemoglobin A1c
HDL-C: High-density lipoprotein-cholesterol
hsCRP: High-sensitivity C-reactive protein
rpm: Revolutions per minute
ELISAs: Enzyme-linked immunosorbent assays
SPSS: Statistical package for social sciences
